# LINC01006 facilitates cell proliferation, migration and invasion in prostate cancer through targeting miR-34a-5p to up-regulate DAAM1

**DOI:** 10.1186/s12935-020-01577-1

**Published:** 2020-10-19

**Authors:** Enhui Ma, Qianqian Wang, Jinhua Li, Xinqi Zhang, Zhenjia Guo, Xiaofeng Yang

**Affiliations:** 1 Department of Urology, Southwest Shandong Hospital Co., Ltd, Liaocheng, 252300 Shandong China; 2 Department of Nephrology, Zaozhuang Municipal Hospital, Zaozhuang, 277100 Shandong China; 3 Orthopeadic Surgery, Southwest Shandong Hospital Co., Ltd, Liaocheng, 252300 Shandong China; 4 Department of Urology, Shandong Zibo Mining Group Central Hospital, Zibo, 255120 Shandong China; 5 Department of Urology, Zaozhuang Municipal Hospital, NO.41 Longtou Road, Shizhong District, Zaozhuang, 277100 Shandong China

**Keywords:** LINC01006, miR-34a-5p, DAAM1, Prostate cancer

## Abstract

**Background:**

Prostate cancer (PCa) is a kind of malignancy occurring in the prostate gland. Substantial researches have proved the major role of long noncoding RNAs (lncRNAs) in PCa. However, the role of long intergenic non-protein coding RNA 1006 (LINC01006) in PCa has not been investigated yet.

**Methods:**

RT-qPCR was used to examine the expression levels of LINC01006 and its downstream targets. The function of LINC01006 in PCa was tested by in vitro and in vivo assays. With application of RNA pull down, RNA immunoprecipitation (RIP) and luciferase reporter assays, the interaction among LINC01006, miR-34a-5p and disheveled associated activator of morphogenesis 1 (DAAM1) were verified.

**Results:**

LINC01006 expression presented high in PCa cell lines. LINC01006 silencing suppressed cell proliferative, migratory, invasive capacities while accelerated apoptotic rate. Besides, LINC01006 knockdown also suppressed tumor growth and metastasis in vivo. Furthermore, miR-34a-5p, a tumor suppressor in PCa, was sponged by LINC01006. Moreover, DAAM1 was targeted by miR-34a-5p and promoted PCa progression. More intriguingly, rescue assays suggested that the inhibitory effect of LINC01006 knockdown on PCa development was offset by DAAM1 overexpression.

**Conclusions:**

LINC01006 promoted PCa progression by sponging miR-34a-5p to up-regulate DAAM1, providing a novel target for PCa therapy.

## Background

Prostate cancer (PCa) is recognized as a type of malignancy for elderly males all over the world [1]. PCa ranks as the third most common deadly cancer on account of its high morbidity and mortality [2, 3]. At the same time, lack of biomarkers in early stage results in poor diagnosis of PCa. Thus, delving into the potential molecular mechanism in PCa for a deep understanding is of vital importance.

Long noncoding RNAs (lncRNAs) are a group of transcripts greater than 200 nts in length with no protein-coding capacity [4]. For the last few years, quantities of lncRNAs have been reported to be crucial regulators in cellular processes of various cancers [5–7]. For instance, a novel lncRNA LSAMP-AS1 is involved in PCa process via targeting miR-183-5p/DCN axis [8]. LncRNA RHPN1-AS1 has been claimed to accelerate the breast cancer development [9]. UCA1/miR-495/PRL-3 axis results in cell proliferation in gastric cancer [10]. LINC01006 was claimed to be a potential biomarker in gastric cancer [11]. However, the mechanism of LINC01006 in the progression of PCa remains largely unknown.

Plentiful researches have proved that lncRNAs act as a competing endogenous RNA (ceRNA) to get involved in cancer. In brief, lncRNAs can serve as sponges for miRNAs to modulate mRNAs [12, 13]. In other words, lncRNAs release mRNAs by binding with miRNAs. For instance, MAGI2-AS3 correlates with NSCLC through sponging miR-155 to regulate SOCS-1 [14]. TTN-AS1 acts as an oncogene in ovarian cancer through enhancing the expression of ROCK2 [15]. In this paper, we focused on determining the ceRNA regulatory mechanism of LINC01006 in PCa. The detailed roles of LINC01006, miR-34a-5p and DAAM1 were assessed by functional assays. Moreover, the relative mechanism was investigated. Rescue assays were carried out to validate the function of LINC01006/miR-34a-5p/DAAM1 axis in PCa.

## Methods

### Cell lines

Human normal cell line (RWPE-1) and four human PCa cell lines, including DU145, PC3, LNCAP and VCaP, were all obtained from ATCC (Manassas, VA). All cells were cultured under 37 °C in 5% CO_2_ in DMEM culture medium (Gibco) containing 10% FBS (Life Technologies, Carlsbad, CA).

### Total RNA extraction and quantitative real-time polymerase chain reaction (RT-qPCR)

Using TRIzol Reagent (Invitrogen, USA), total RNA was isolated and cDNA was synthesized by PrimeScript RT master mix (Takara, Tokyo, Japan). To evaluate the expression of genes, SYBR Green PCR Kit (Takara) was applied to conduct qPCR. The quantitative analysis was determined by 2^−ΔΔCt^ method and all results were normalized to U6 or GAPDH. The experiments were performed at least three times.

### Plasmid construct and transfection

The designed shRNAs targeting LINC01006 or DAAM1, as well as their corresponding control shRNA plasmids, were all synthesized by Genechem (Shanghai, China). Besides, pcDNA3.1 vectors (Invitrogen) were subcloned with DAAM1 or LINC01006 for overexpression, and empty pcDNA3.1 vector was used as the negative control. Mimics/inhibitor of miR-34a-5p and NC mimics/inhibitor were procured from RiboBio (Shanghai, China). All cell transfection were performed using Lipofectamine 3000 (Invitrogen).

### Colony formation assay

Cells were seeded into 6-well plates at a density of 500 cells per well for 2 weeks of incubation. After 30 min fixation with 4% PFA, 0.5% crystal violet solution was used for cell staining for 5 min. In the end, the visible colonies were manually recorded. The experiments were performed at least three times.

### Ethynyl-2-deoxyuridine (EdU) incorporation assay

Using BeyoClick™ EdU Cell Proliferation Kit (Beyotime, Shanghai, China) with Alexa Fluor 594, EdU proliferation assay was conducted. In brief, transfected cells were cultured in 96-well plates with 1 × 10^4^ cells/well. After double-stained using EdU and DAPI solution, cells were finally observed under laser confocal microscopy (Olympus, Tokyo, Japan). The experiments were performed at least three times.

### TUNEL assay

In brief, transfected DU145 and LNCAP cells were treated with 4% PFA and then added with TUNEL reagent (Merck KGaA, Darmstadt, Germany). After being stained with DAPI dye, optical microscopy (Olympus) was used to analyze labeled samples. The experiments were performed at least three times.

### Flow cytometry

Double Annexin V/PI staining kit (Invitrogen) was applied to estimate cell apoptosis. After 15 min of double-staining with binding buffer, cell samples were then determined with a flow cytometer (FACScan, BD Biosciences). The experiments were performed at least three times.

### Transwell assay

In brief, 2 × 10^4^ cells were plated into the upper chambers of transwell plates. Then the bottom chamber was added with 100% complete culture medium. The chambers were added with Matrigel (BD Biosciences San Diego, CA, USA) for invasion assay and no Matrigel for migration assay. 24 h later, cells were stained by crystal violet for counting. The experiments were performed at least three times.

### Western blot analysis

Total protein samples were separated with SDS-PAGE and later transferred to PVDF membranes (Thermo fisher, IL, USA). Afterwards, membranes went through incubation with primary antibodies at 4℃ overnight after being blocked in milk without fat. After washed with PBS × 3, the blots were then incubated with secondary antibody for 1 h at dark room. The antibodies against DAAM1 and β-actin were bought from Santa Cruz Biotechnology (Dallas, USA). The quantification analysis of protein was conducted by Chemiluminescence system (GE Healthcare, Chicago, USA). The experiments were performed at least three times.

### Subcellular fractionation

Subcellular fractionation assay was carried out with PARIS™ Kit (Invitrogen) to isolate the nuclear and cytoplasmic RNAs from PCa cells. GAPDH was treated as cytoplasmic control for quantification, while U6 was the nuclear control. The experiments were performed at least three times.

### FISH

After fixation with 4% PFA for 15 min at 37 °C and then permeabilized with 0.5% Triton X-100, PCa cells were hybridized with LINC01006-specific FISH probe in buffer, followed by counterstaining with Hoechst solution. With a confocal laser microscope (Olympus), images were obtained. The experiments were performed at least three times.

### Luciferase reporter assay

Fragments of LINC01006 or DAAM1 containing wild-type and mutant binding sites of miR-34a-5p were cloned into the pmirGLO dual-luciferase vector (Promega, Madison, WI) to respectively construct LINC01006-Wt/Mut and DAAM1-Wt/Mut. DU145 and LNCAP cells were co-transfected with above-mentioned plasmids and miR-34a-5p mimics or NC mimics. Luciferase activity was analyzed using dual-luciferase reporter assay system (Promega). The experiments were performed at least three times.

### RNA pull down

MiR-34a-5p wild type or mutant type sequence was labeled with biotin. Then, biotin-labeled RNA was bound to the magnetic beads (Invitrogen). The bound RNAs were purified for RT-qPCR analysis. The experiments were performed at least three times.

### RNA immunoprecipitation (RIP)

Using RNA-Binding Protein Immunoprecipitation Kit (Millipore, Burlington, MA, USA), RIP assay was performed with control IgG antibody or human Ago2 antibody. After lysed in RIPA buffer, cell lysates were mixed with antibody-bound magnetic beads. Subsequently, the immunoprecipitated RNAs were evaluated by RT-qPCR analysis. The experiments were performed at least three times.

### Animal experiments

BALB/c nude mice (at age of 5–6 weeks) were acquired from SLRC Laboratory Animals (Shanghai, China). For the in vivo tumor formation assay, DU145 cells transfected with sh-NC and sh-LINC01006#1 were suspended in PBS. After that, cells were subcutaneously injected into mice. The tumor volume was measured every 5 days. After 1 month, the mice were euthanized. Then mice tumors were excised and weighed.

For the tumor metastasis assay, DU145 cells transfected with sh-NC and sh-LINC01006#1 were injected into the tail vein of nude mice. The mice were euthanized 50 days later. Then the lungs were removed and fixed with dampen formaldehyde solution for hematoxylin–eosin (H&E) staining. Finally, the lung metastases were observed by microscope. All animal studies were approved by the Ethic committee of Southwest Shandong Hospital Co., Ltd. The experiments were performed at least three times.

### Statistical analyses

All experimental data of three individually performed experiments were presented as the mean ± SD and analyzed by GraphPad Prism 7 (GraphPad Software, Inc., La Jolla, CA). Student’s t-test or one-way ANOVA analysis was used to analyze differences between groups. The experiments were conducted at least three times. The experimental results were considered statistically significant when p value was less than 0.05.

## Results

### LINC01006 is dramatically up-regulated and facilitates the progression of PCa cells

**Fig. 1:**
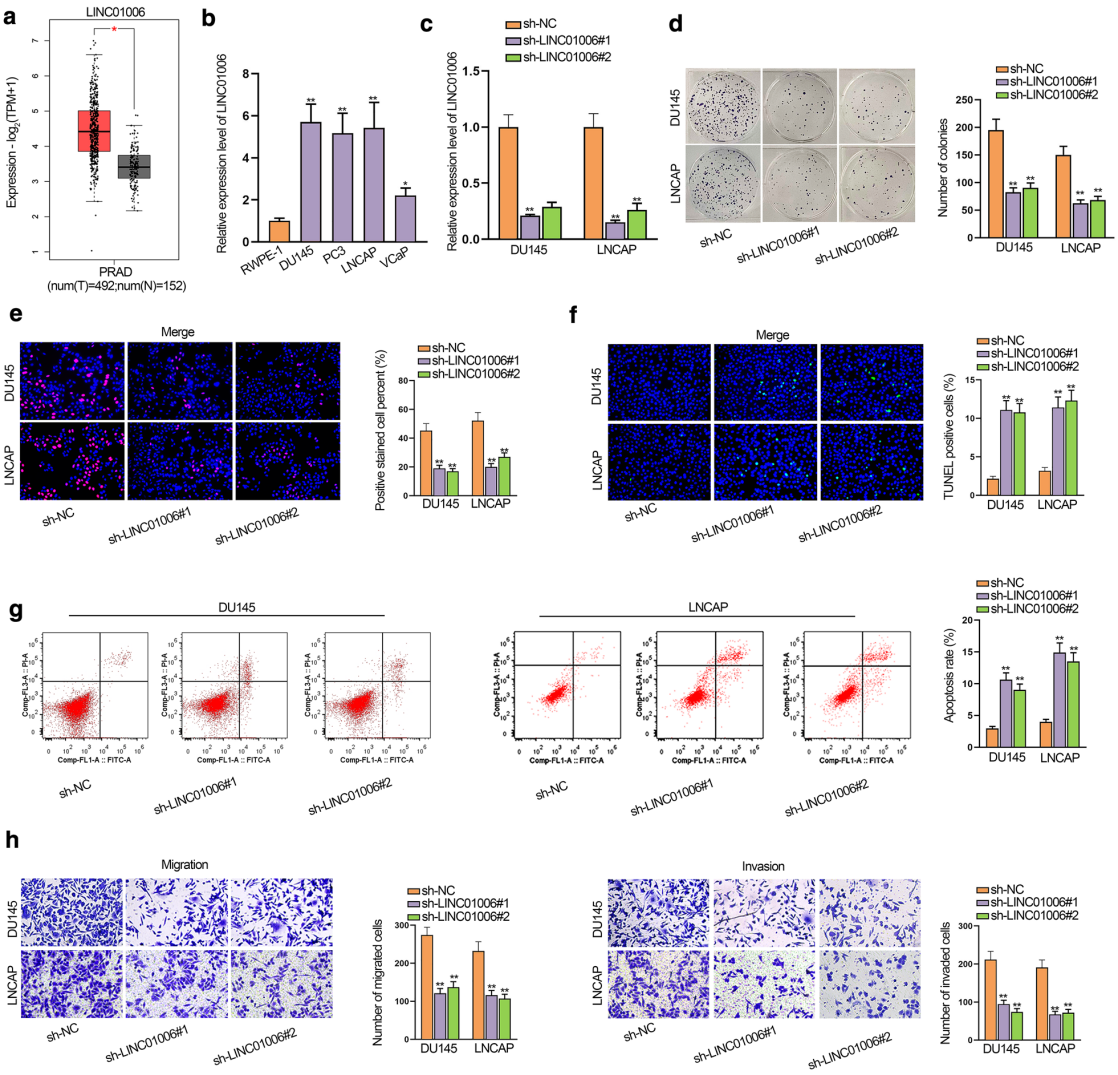
LINC01006 is dramatically up-regulated and facilitates the progression of PCa cells. **a** GEPIA data displayed that LINC01006 was up-regulated in PCa tissues. **b** The expression of LINC01006 in PCa cell lines and normal cell line was detected by RT-qPCR. **c** The knockdown efficiency of sh-LINC01006#1/2 was examined by RT-qPCR. **d**, **e** Colony formation and EdU assays were performed to appraise proliferation in DU145 and LNCAP cells. **f**, **g** Apoptosis rate was examined by TUNEL assay and flow cytometry analysis in different groups. **h** Transwell assays were performed to assess cell migration and invasion. *P < 0.05; **P < 0.01

Firstly, we analyze the expression levels of LINC01006 from GEPIA database (https://gepia.cancer-pku.cn/). As shown in Fig. 1a, LINC01006 exhibited a significant difference in PCa tissues and adjacent non-cancerous tissues. Meanwhile, with the normal human prostate epithelial cell line (RWPE-1) as a control, we validated the up-regulation of LINC01006 in PCa cell lines (DU145, PC3, LNCAP and VCaP) (Fig. 1b). To better understand the role of LINC01006 in PCa, we firstly effectively knocked down LINC01006 in PCa cells (Fig. 1c). Through colony formation and EdU assays, we observed a significant decrease in cell proliferation after LINC01006 silencing (Fig. 1d, e). In contrast, TUNEL and flow cytometry analysis demonstrated that down-regulation of LINC01006 prominently promoted cell apoptosis (Fig. 1f, g). Moreover, the results of transwell assays exhibited the dramatically reduced capacity to migrate and invade in LINC01006-deficient DU145 and LNCAP cells (Fig. 1h). In conclusion, LINC01006 was obviously elevated and promoted cell proliferation, migration and invasion in PCa.

### Knockdown of LINC01006 inhibits tumorigenesis and metastasis in vivo

**Fig. 2:**
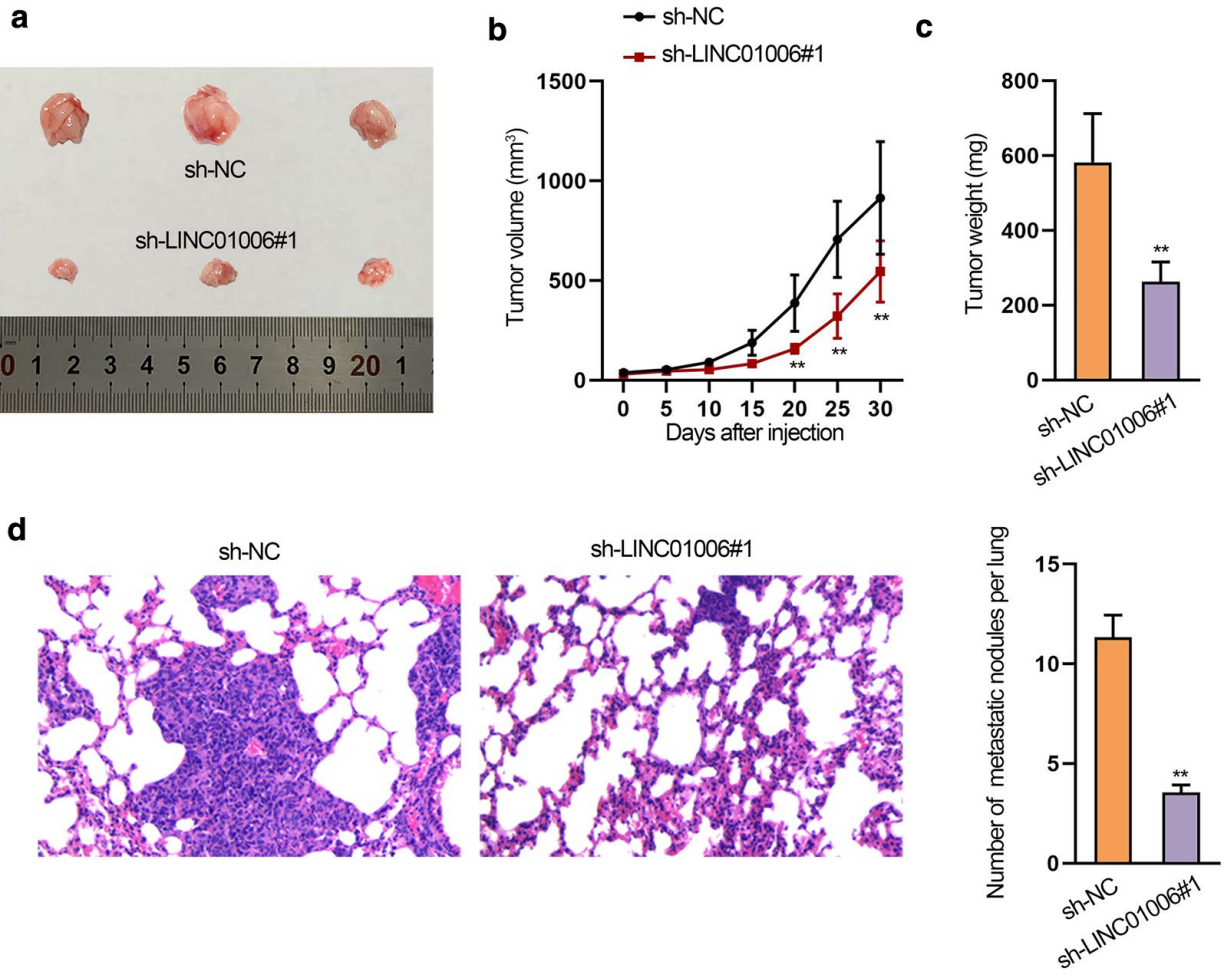
Knockdown of LINC01006 inhibits tumorigenesis and metastasis in vivo. **a** Representative pictures of tumors in sh-NC and sh-LINC01006#1 groups. **b** The growth curve of tumors in above two groups. **c** Tumor weight was lowered after LINC01006 knockdown in comparison with that in the control group. **d** Images obtained via HE staining showed lung metastatic nodules in sh-NC or sh-LINC01006#1 group (*left panels*). Differences of lung metastasis nodules between these two groups were assessed by quantitative analysis (*right panels*). *P < 0.05; **P < 0.01

To further elucidate the function of LINC01006 in PCa tumorigenesis and metastasis in vivo, DU145 cells transfected with sh-NC and sh-LINC01006#1 were injected into 6-week-old nude mice. As expected, compared with the control group, there was a much smaller tumor size in the sh-LINC01006#1 group (Fig. 2a). Similarly, the tumor growth rate became slower after knockdown of LINC01006 (Fig. 2b). Not surprisingly, tumor weight in sh-LINC01006#1 group was also significantly lowered compared to that in control group (Fig. 2c). At the same time, we constructed a lung metastasis model. The results showed that the number of metastatic nodules was markedly reduced in sh-LINC01006#1 group (Fig. 2d), indicating that metastasis in vivo was repressed when LINC01006 was silenced. Therefore, knockdown of LINC01006 suppressed PCa tumorigenesis and metastasis in vivo.

### MiR-34a-5p is sponged by LINC01006 and inhibits the development of PCa

**Fig. 3:**
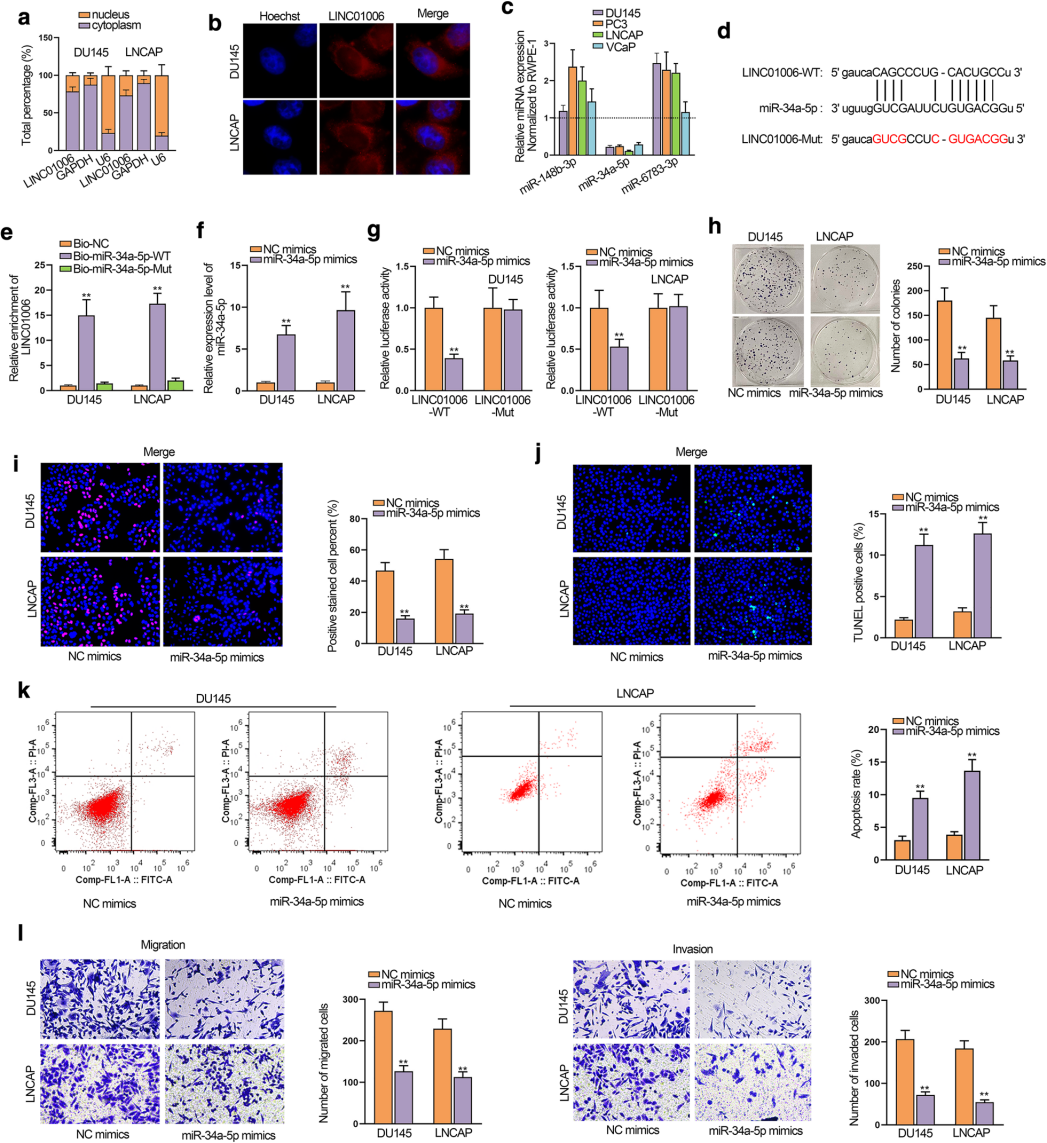
MiR-34a-5p is sponged by LINC01006 and inhibits the development of PCa. **a**, **b** Nucleus-cytoplasm fractionation and FISH assays were carried out to judge subcellular localization of LINC01006. **c** RT-qPCR measured the expression of 3 miRNAs in PCa cell lines. **d** Binding sites between miR-34a-5p and LINC01006. **e** RNA pull down assays were performed to validate the interaction between miR-34a-5p and LINC01006. **f** RT-qPCR was used to evaluate miR-34a-5p expression. **g** Luciferase reporter assays were carried out to examine the interaction between miR-34a-5p and LINC01006. **h**, **i** Cell proliferation was assessed via colony formation and EdU assays in cells transfected with miR-34a-5p mimics. **j**, **k** Apoptosis rate was appraised by TUNEL assay and flow cytometry analysis after overexpression of miR-36a-5p. **l** Transwell assays evaluated cell migratory and invasive abilities after miR-34a-5p upregulation. **P < 0.01

Thereafter, we intended to determine the subcellular localization of LINC01006 for further investigation of the mechanism involved in PCa cells. As shown in Fig. 3a, b, the abundant accumulation of LINC01006 in cytoplasm was observed. Thus, we speculated that LINC01006 might function as a ceRNA to regulate targeted mRNAs via sponging certain miRNAs [16]. To predict miRNAs binding to LINC01006, we used starBase (https://starbase.sysu.edu.cn/) and selected out 3 miRNAs (miR-148b-3p, miR-34a-5p and miR-6783-3p) on the condition of CLIP-Data ≥ 1, Degradome-Data ≥ 0, and Pan-Cancer ≥ 6. Among these miRNAs, miR-34a-5p was the only one that exhibited down-regulated expression in all the four PCa cells (Fig. 3c). Hence, we presumed that LIN01006 was a sponge for miR-34a-5p, and Fig. 3d showed the predictive binding sites between them. For further confirmation of the correlation between LINC01006 and miR-34a-5p, RNA pull down assay was implemented and the results uncovered that LINC01006 could be pulled down by biotinylated miR-34a-5p-WT in PCa cells (Fig. 3e). To increase miR-34a-5p expression, we transfected miR-34a-5p mimics into PCa cells (Fig. 3f). The outcomes of luciferase reporter assays corroborated that the luciferase activity of LINC01006-WT instead of LINC01006-Mut was obviously lessened by miR-34a-5p mimics (Fig. 3g). At the same time, knockdown of LINC01006 couldn’t affect miR-34a-5p expression (Additional file 1: Fig. S1A). Furthermore, up-regulation of LINC01006 had no influence on miR-34a-5p level, either (Additional file 1: Fig. S1B, C). These results all suggested that LINC01006 sponged miR-34a-5p instead of regulating miR-34a-5p. After that, functional assays revealed that cell proliferation was repressed by miR-34a-5p overexpression (Fig. 3h, i). On the contrary, miR-34a-5p overexpression enhanced the rate of cell apoptosis (Fig. 3j, k). Migratory and invasive capacities detected by transwell assays were also prevented by miR-34a-5p mimics (Fig. 3l). However, changes in phenotypes induced by miR-34a-5p mimics were all rescued by overexpression of LINC01006 (Additional file 1: Fig. S1D–I). To sum up, miR-34a-5p was sponged by LINC01006 and moderated PCa progression.

### DAAM1 is a target gene of miR-34a-5p

**Fig. 4:**
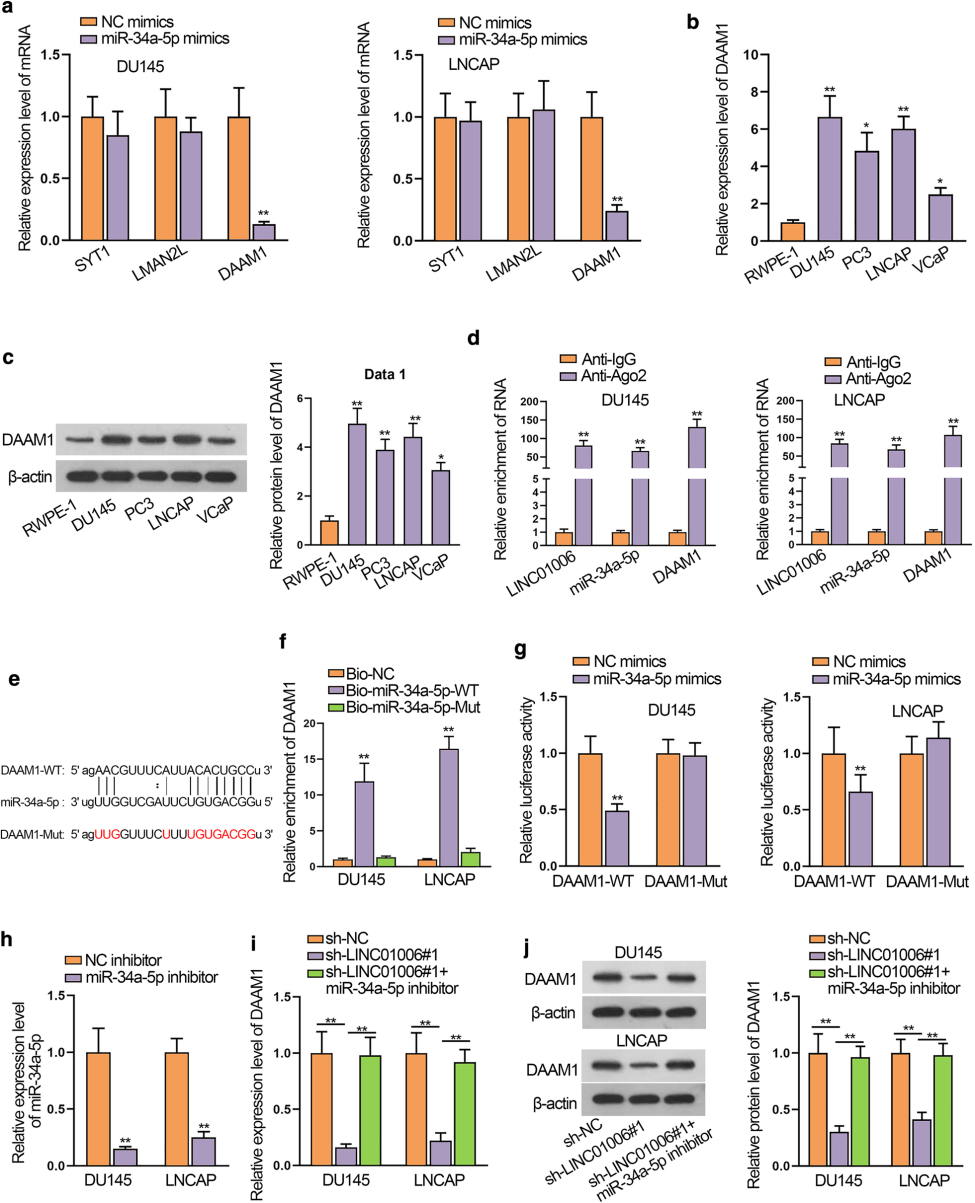
DAAM1 is a target gene of miR-34a-5p. **a** RT-qPCR evaluated the expression of 3 mRNAs when miR-34a-5p was up-regulated. **b** DAAM1 mRNA expression was detected in PCa cells and normal RWPE-1 cells by RT-qPCR. **c** Protein levels of DAAM1 were determined by western blot. **d** RIP assay was performed to validate that LINC01006, miR-34a-5p and DAAM1 were coexisted in Ago2 pallets. **e** Bioinformatics tools predicted the binding sites between miR-34a-5p and DAAM1. **f**, **g** Interaction between miR-34a-5p and DAAM1 at above binding sites was certified by RNA pull down and luciferase reporter assays. **h** RT-qPCR measured miR-34a-5p expression when cells were transfected with miR-36a-5p inhibitor. **i** DAAM1 mRNA expression was determined by RT-qPCR in sh-NC, sh-LINC01006#1 and sh-LINC01006#1 + miR-34a-5p mimics groups. **j** DAAM1 protein levels were also detected by western blot in sh-NC, sh-LINC01006#1 and sh-LINC01006#1 + miR-34a-5p mimics groups. *P < 0.05; **P < 0.01

Next, we concentrated on exploring the target gene of miR-34a-5p. By means of starBase, SYT1, LMAN2L and DAAM1 were predicted to have binding sites for miR-34a-5p. Data of RT-qPCR analysis supported that up-regulation of miR-34a-5p could only lower the expression of DAAM1 rather than SYT1 or LMAN2L in PCa cells (Fig. 4a). Besides, DAAM1 mRNA level and protein level were determined to be elevated in PCa cells (Fig. 4b, c). Then, RIP assays were carried out and it turned out that LINC01006, miR-34a-5p and DAAM1 were preferentially concentrated in the Ago2 precipitates (Fig. 4d). By the prediction of bioinformatics analysis, the binding sites between miR-34a-5p and DAAM1 were shown in Fig. 4e. The interaction between DAAM1 and miR-34a-5p was also validated by RNA pull down assay (Fig. 4f). Luciferase reporter assay disclosed that miR-34a-5p mimics diminished the luciferase activity of DAAM1-WT while DAAM1-Mut luciferase activity showed no apparent change (Fig. 4g). Later, we cut down the expression of miR-34a-5p by transfection of miR-34a-5p inhibitor (Fig. 4h). More intriguingly, we observed that miR-34a-5p inhibitor restored the decreased DAAM1 expression generated by LINC01006 silence (Fig. 4i). Similarly, western blot analysis showed that knockdown of LINC01006 in DU145 and LNCAP cells triggered a significant silencing effect on endogenous DAAM1 protein expression, while miR-34a-5p inhibitor reversed such effect (Fig. 4j). Collectively, DAAM1 was targeted by miR-34a-5p and highly expressed in PCa cells.

### DAAM1 facilitates PCa progression

**Fig. 5:**
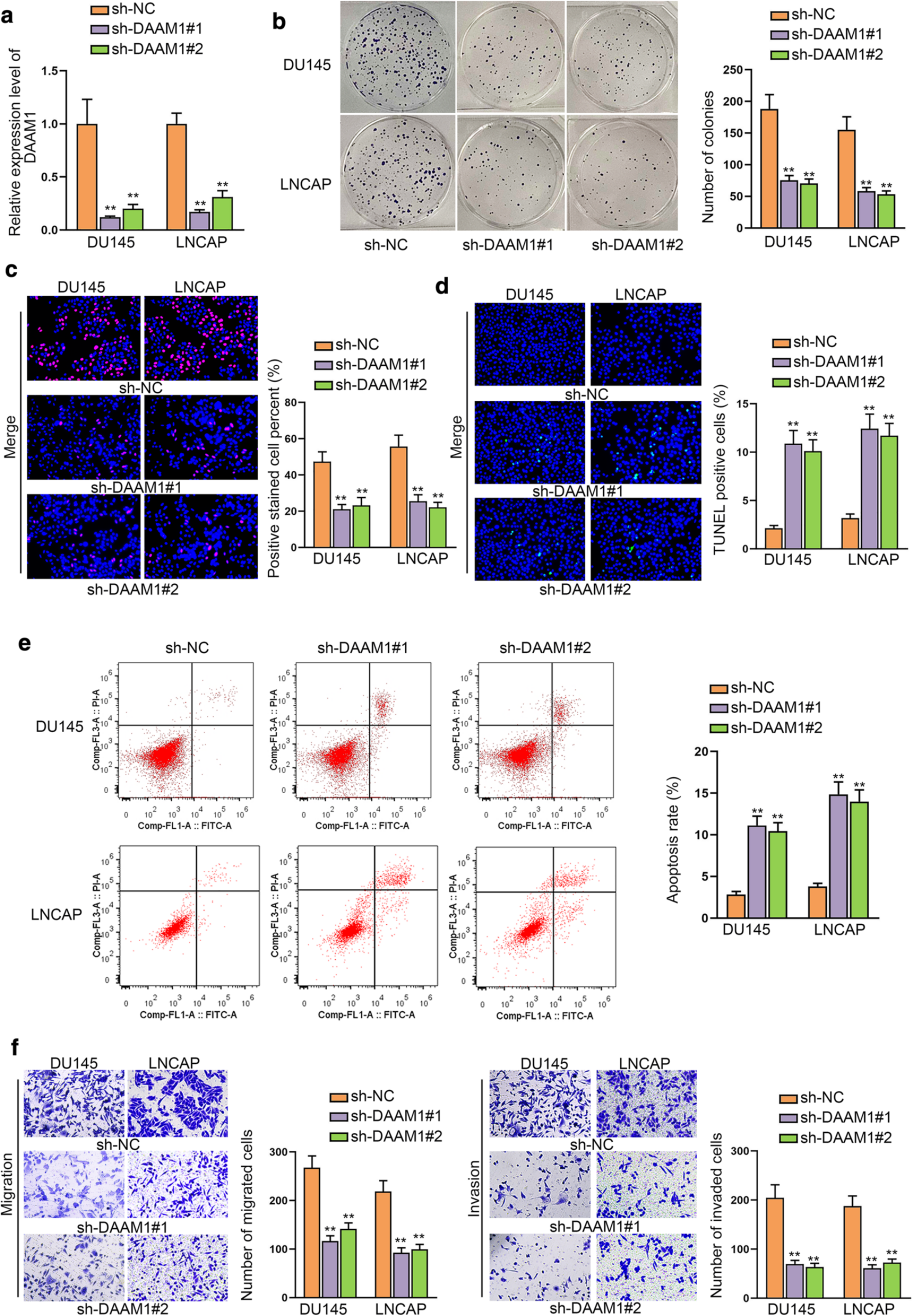
DAAM1 facilitates PCa progression. **a** RT-qPCR examined sh-DAAM1#1/2 efficiency in PCa cells. **b**, **c** Cell proliferation was assessed via colony formation and EdU assays. **d**, **e** Apoptosis rate was appraised by TUNEL assay and flow cytometry analysis. **f** Migratory and invasive abilities were examined by transwell assay. **P < 0.01

In addition, the influence of DAAM1 on cell proliferation, migration, invasion and apoptosis was further investigated by functional assays. In the beginning, sh-DAAM1#1 and sh-DAAM1#2 were transfected to lessen the expression of DAAM1 (Fig. 5a). As shown in Fig. 5b, c, cell proliferation was repressed by DAAM1 knockdown. In contrast, DAAM1 down-regulation enhanced the apoptosis rate (Fig. 5d, e). Meanwhile, transwell assays also revealed that DAAM1 depletion suppressed cell migration and invasion (Fig. 5f). To sum up, DAAM1 facilitated cell proliferation, migration, invasion and inhibited cell apoptosis in PCa.

### LINC01006 promotes the progression of PCa by regulating DAAM1

**Fig. 6:**
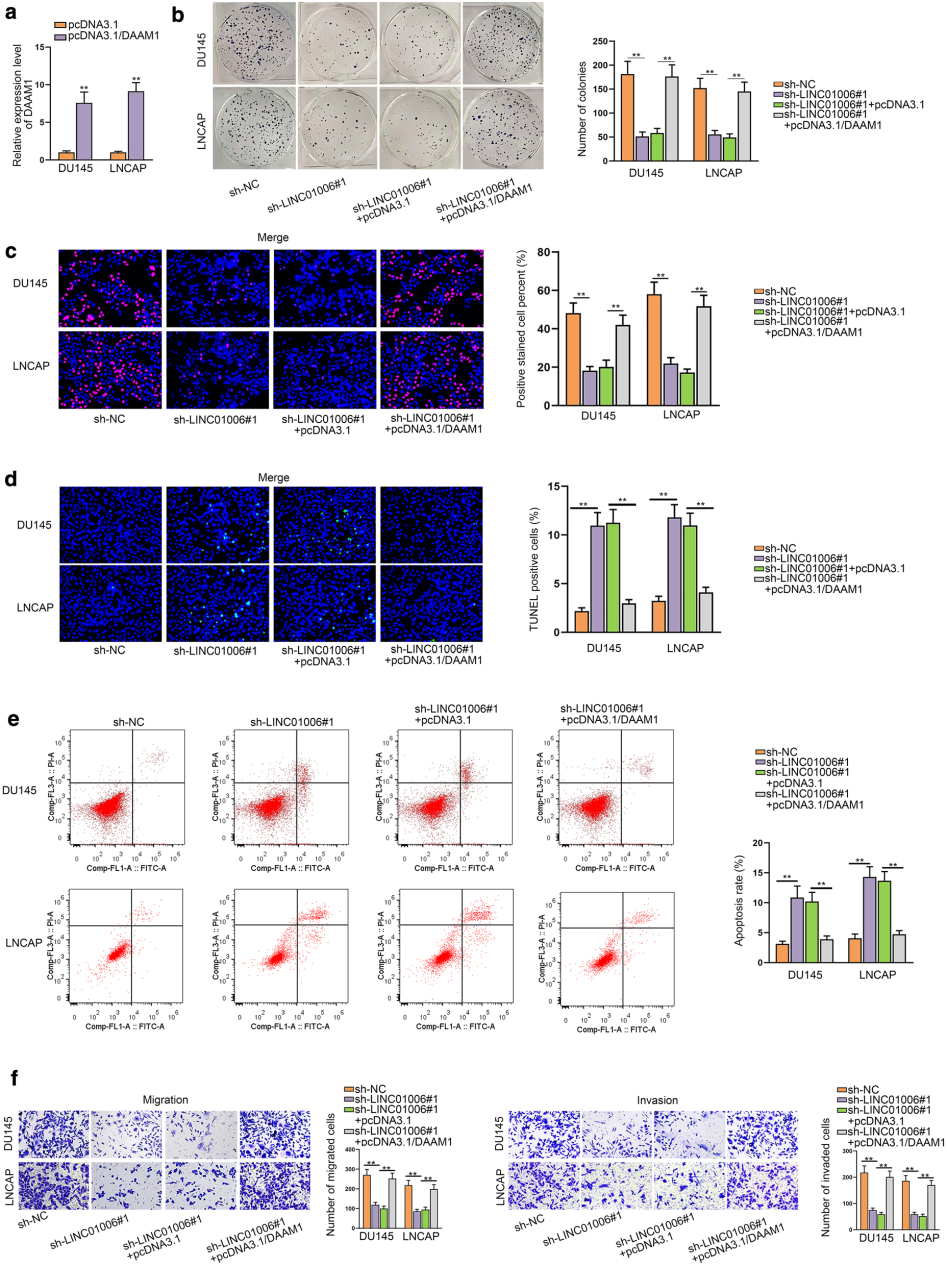
LINC01006 promotes the progression of PCa by regulating DAAM1. **a** RT-qPCR measured DAAM1 expression in cells transfected with pcDNA3.1/DAAM1. **b**, **c** Colony formation and EdU assays were conducted to examine cell proliferation in cells with sh-NC, sh-LINC01006#1, sh-LINC01006#1 + pcDNA3.1 and sh-LINC01006#1 + pcDNA3.1/DAAM1 groups. **d**, **e** TUNEL assay and flow cytometry analysis were performed to observe cell apoptosis in different groups. **f** Transwell assays assessed cell migration and invasion in different groups. **P < 0.01

To affirm that LINC01006 up-regulated DAAM1 to facilitate PCa progression, rescue assays were performed. Firstly, pcDNA3.1/DAAM1 was constructed and transfected into cells to elevate DAAM1 expression (Fig. 6a). Thereafter, it manifested that the descending proliferation capacity due to LINC01006 knockdown was rescued by DAAM1 up-regulation (Fig. 6b, c). Also, the results of TUNEL assay and flow cytometry analysis demonstrated that the promoted cell apoptosis imposed by LINC01006 down-regulation was offset by DAAM1 overexpression (Fig. 6d-e). In the meantime, DAAM1 overexpression counteracted the descending tendency of migratory and invasive capacity induced by silencing of LINC01006 (Fig. 6f). On all accounts, LINC01006 enhanced DAAM1 expression to promote PCa development.

## Discussion

Aberrant expression of lncRNAs has been reported to affect the development of cancers [17, 18]. For example, PCGEM1/miR-433-3p/FGF2 signaling pathway contributes to malignant phenotypes of renal carcinoma cells [19]. PVT1 functions as a tumor promoter in NSCLC via sequestering miR-526b to modulate EZH2 [20]. Up-regulation of OIP5-AS1 boosts the progression of gastric cancer [21]. LINC01006 is certified to promote pancreatic cancer development through functioning as a ceRNA of miR-2682-5p to enhance HOXB8 [22]. Through our investigation, LINC01006 was determined to be notably overexpressed and stimulate the development of PCa. All the findings testified the oncogenic role of LINC01006 in PCa.

A large number of researches have manifested that ceRNA network is a potential mechanism when we analyze the function of lncRNAs in diseases [23]. For example, BCAR4 aggravates bladder cancer development via functioning as a miR-370-3p sponge to activate Wnt signaling pathway [24]. Through miR-139/PIK3CA activated PI3k/Akt signaling pathway, LINC00152 accelerates tumorigenesis in hepatocellular carcinoma [25]. In this study, we firstly identified the ceRNA mechanism of LINC01006 in PCa. Moreover, miR-34a-5p was found out to be sponged by LINC01006. As a matter of fact, miR-34a-5p has been verified to hinder the migration and invasion of human epidermal keratinocytes by a previous study [26]. Likewise, our research suggested that miR-34a-5p could repress PCa development.

Mounting assays highlight that mRNAs are regulated by miRNAs [27, 28]. In our study, miR-34a-5p was validated to target DAAM. Besides, DAAM1 was obviously up-regulated in PCa cell lines. The finding that DAAM1 up-regulation was connected with cell migration and invasion in ovarian cancer has been displayed by a former research [29]. In the functional assays, we also found that DAAM1 down-regulation inhibited tumorigenesis of PCa. Eventually, a list of rescue assays substantiated that the regulatory effect of LINC01006 silence on cell proliferation, apoptosis, migration and invasion of PCa could be restored by DAAM1 up-regulation.

## Conclusion

Overall, via sequestering miR-34a-5p and enhancing DAAM1 expression, LINC01006 motivated PCa development, suggesting that LINC01006 could act as a potential biomarker for PCa.

## Supplementary information

**Additional file 1: Figure S1.** The effect of knockdown or overexpression of LINC01006 on miR-34a-5p expression and function (A) RT-qPCR measured the expression level of miR-34a-5p when LINC01006 was silenced. (B) RT-qPCR validated that pcDNA3.1/LINC01006 was transfected into VCaP cells to enhance the expression level of LINC01006. (C) RT-qPCR measured the expression level of miR-34a-5p when LINC01006 was overexpressed in VCaP cells. (D-E) Colony formation and EdU assays were carried out to evaluate cell proliferation in different groups. (F-G) TUNEL assay and flow cytometry analysis were performed to detect cell apoptosis rate in different groups. (H-I) Transwell assay was applied to assess migratory and invasive capacities in DU145 and LNCAP cells. **P < 0.01.

## Data Availability

Not applicable.

## Abbreviations

LINC01006: Long intergenic non-protein coding RNA 1006
DAAM1: Dishevelled associated activator of morphogenesis 1
lncRNAs: Long non-coding RNAs
ceRNAs: Competing endogenous RNAs
PCa: Prostate cancer
miRNAs: MicroRNAs
mRNA: Messenger RNA
ATCC: American type culture collection
DMEM: Dulbecco’s modified Eagle’s medium
FBS: Fetal bovine serum
RIPA: Radioimmunoprecipitation assay
SDS-PAGE: Sulphate–polyacrylamide gel electrophoresis
PVDF: Polyvinylidene fluoride
DAPI: 4′,6-Diamidino-2-phenylindole
RT-qPCR: Quantitative real-time polymerase chain reaction
EdU: Ethynyl-2-deoxyuridine
TUNEL assay: Terminal deoxynucleotidyl transferase-mediated dUTP-biotin nick end labeling assay
FISH: Fluorescence in situ hybridization
RIP: RNA immunoprecipitation
H&E staining: Hematoxylin and eosin staining
wild-type: WT
mutant: Mut
SD: Standard deviation
ANOVA: Analysis of variance
