# Supplementary figures and images for: LINC01006 facilitates cell proliferation, migration and invasion in prostate cancer through targeting miR-34a-5p to up-regulate DAAM1
Source: Cancer Cell Int. 2020 Oct 19;20:515. doi: 10.1186/s12935-020-01577-1 (PMC7574442; doi:10.1186/s12935-020-01577-1)

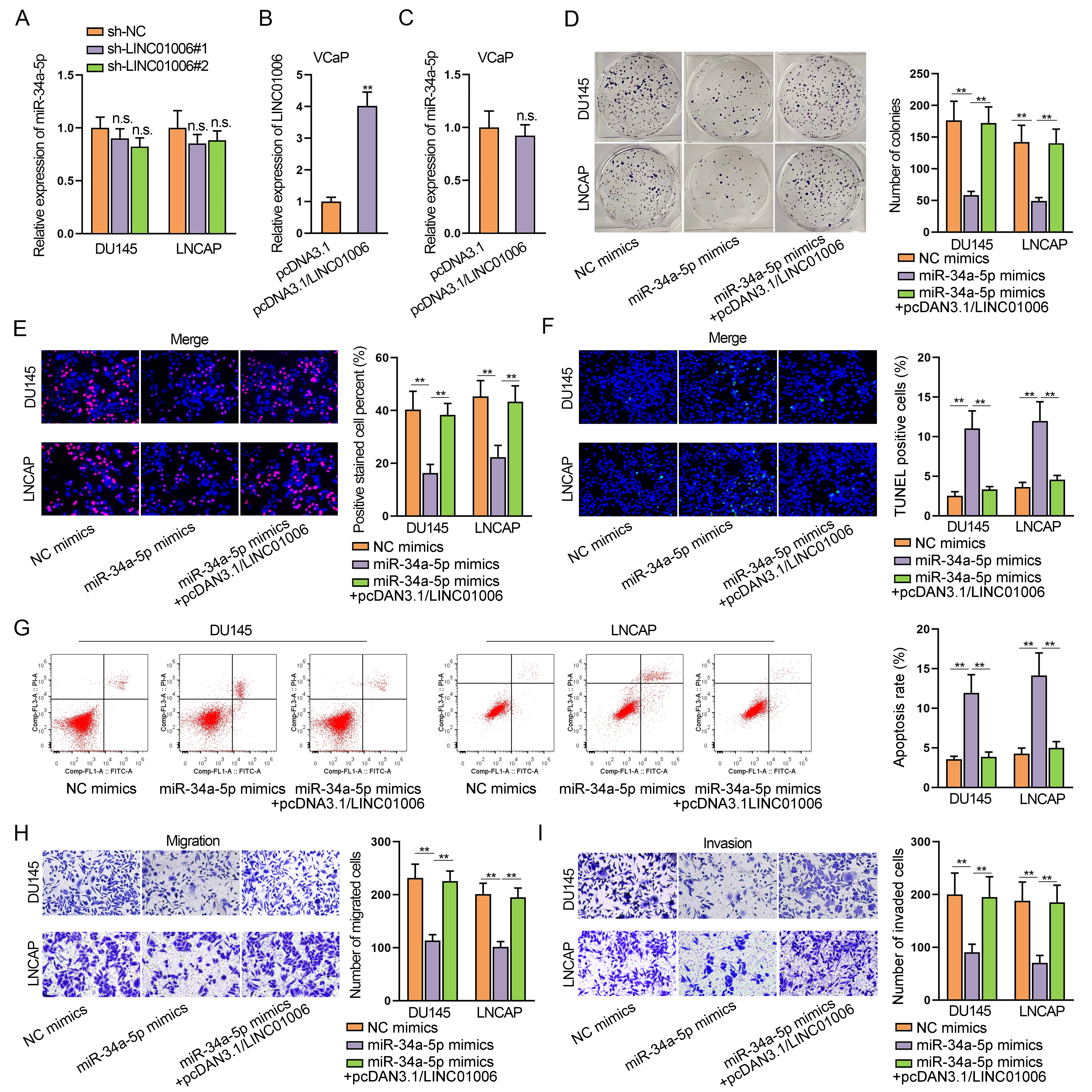

Supplement: Supplementary file 1 — Additional file 1: Figure S1. The effect of knockdown or overexpression of LINC01006 on miR-34a-5p expression and function (A) RT-qPCR measured the expression level of miR-34a-5p when LINC01006 was silenced. (B) RT-qPCR validated that pcDNA3.1/LINC01006 was transfected into VCaP cells to enhance the expression level of LINC01006. (C) RT-qPCR measured the expression level of miR-34a-5p when LINC01006 was overexpressed in VCaP cells. (D-E) Colony formation and EdU assays were carried out to evaluate cell proliferation in different groups. (F-G) TUNEL assay and flow cytometry analysis were performed to detect cell apoptosis rate in different groups. (H-I) Transwell assay was applied to assess migratory and invasive capacities in DU145 and LNCAP cells. **P < 0.01. [file 12935_2020_1577_MOESM1_ESM.tif]
